# In Situ Sea Cucumber Detection across Multiple Underwater Scenes Based on Convolutional Neural Networks and Image Enhancements

**DOI:** 10.3390/s23042037

**Published:** 2023-02-10

**Authors:** Yi Wang, Boya Fu, Longwen Fu, Chunlei Xia

**Affiliations:** 1 Coastal Defense College, Naval Aeronautical University, Yantai 264003, China; 2 Yantai Institute of Coastal Zone Research, Chinese Academy of Sciences, Yantai 264003, China

**Keywords:** marine organism recognition, marine survey, YOLOv7, YOLOv5, object detection, underwater image enhancement

## Abstract

Recently, rapidly developing artificial intelligence and computer vision techniques have provided technical solutions to promote production efficiency and reduce labor costs in aquaculture and marine resource surveys. Traditional manual surveys are being replaced by advanced intelligent technologies. However, underwater object detection and recognition are suffering from the image distortion and degradation issues. In this work, automatic monitoring of sea cucumber in natural conditions is implemented based on a state-of-the-art object detector, YOLOv7. To depress the image distortion and degradation issues, image enhancement methods are adopted to improve the accuracy and stability of sea cucumber detection across multiple underwater scenes. Five well-known image enhancement methods are employed to improve the detection performance of sea cucumber by YOLOv7 and YOLOv5. The effectiveness of these image enhancement methods is evaluated by experiments. Non-local image dehazing (NLD) was the most effective in sea cucumber detection from multiple underwater scenes for both YOLOv7 and YOLOv5. The best average precision (AP) of sea cucumber detection was 0.940, achieved by YOLOv7 with NLD. With NLD enhancement, the APs of YOLOv7 and YOLOv5 were increased by 1.1% and 1.6%, respectively. The best AP was 2.8% higher than YOLOv5 without image enhancement. Moreover, the real-time ability of YOLOv7 was examined and its average prediction time was 4.3 ms. Experimental results demonstrated that the proposed method can be applied to marine organism surveying by underwater mobile platforms or automatic analysis of underwater videos.

## 1. Introduction

Sea cucumber (*Stichopus japonicus*) is widely used for food and medicine in Asian and Middle Eastern countries [1]. Recently, sea cucumber fisheries have grown quickly, especially in China [2]. However, the management in sea cucumber fisheries is mostly inefficient, and improving sustainable stock is required worldwide [3,4]. Currently, growth inspection, population surveying and harvesting in sea cucumber aquaculture are conducted by experienced human divers, which is dangerous and not very efficient. 

Recently, advanced underwater robotics and artificial intelligence have provided technical solutions for improving the automation level and decreasing production costs in aquaculture. Visual observation is one of the key techniques for achieving intelligent aquaculture. Optical digital imaging has been rapidly developing in the last decades [5]. High resolution images could be acquired by using low-cost portable cameras. Low-cost portable cameras have become a common tool for marine biodiversity investigation, underwater surveillance and visual inspection tasks. The cameras could be carried by divers or integrated into observation facilities (e.g., remote operated vehicles (ROV) or observation stations) to collect underwater images or videos [6]. 

Numerous underwater videos and digital images have been collected in coastal cultivation areas. Currently, these video and image records should be examined by trained human experts for population surveying or growth inspection in natural conditions. Automatic analysis of sea cucumber in natural conditions has not been widely applied to aquaculture practices. Detecting sea cucumber in its natural habitat is a challenging task due to the complicated illumination conditions, turbidity of water, varying body shape and occlusions. For practical applications in sea cucumber cultivation, automatic sea cucumber detection and monitoring are challenging and necessary. Most studies of sea cucumber image analysis in natural conditions have been made in recent years. Classification of eight species of benthic organisms, including sea cucumber, was implemented by a tree-structured support vector machine (SVM) classifier [7]. Color, texture and structure features were extracted from the target images. The results showed that some taxa (e.g., the small sea cucumber) are still challenging for both humans and observation systems. Movement tracking of sea cucumber by underwater video was implemented by a color histogram mean-shift based tracking [8]. This method was able to track the sea cucumber movement; however, manual initialization is required to select the individual image of sea cucumber to initialize the tracking process. Another limitation was the fact that the tracking algorithm mainly depends on color features. Color distortion issues are severe in underwater conditions and the sea cucumber was not always distinguishable from the natural background. To minimize the color distortion, the underwater images were initially processed by contrast limited adaptive histogram equalization (CLAHE) to enhance the image features [9]. Sea cucumber segmentation is conducted by using an active contour model in natural conditions [10]. The edge feature of thorns was utilized to locate individual sea cucumbers from the underwater images. The active contour models (ACM) demonstrated excellent capability in matching the body shape of the individual organisms [11]. The above works dealt with single sea cucumbers from a specified condition, and the location of the sea cucumber was given in the image.

Accurate detection of individual sea cucumbers is a fundamental initial step toward sophisticated sea cucumber image analysis (e.g., tracking or segmentation). Unfortunately, illumination variations, shadows and occlusions are still the most challenging issues in underwater sea cucumber detection. In recent years, deep neural networks have rapidly developed and been successfully applied to computer vision fields [12]. Generic object detection algorithms, such as faster region-based convolutional neural network (Faster R-CNN), single shot multibox detector (SSD), and you only look once (YOLO), have been applied to the in situ detection of marine organisms (e.g., sea cucumbers) [13,14]. These works focused on common issues suffered in underwater object detection, e.g., real-time detection ability, small target detection and the improvement of detection and recognition accuracy. To improve the real-time detection ability, lightweight models were developed by pruning the existing object detection models (e.g., SDD) or integrating lightweight backbones such as MobileNet [15,16]. On the other hand, multiple scale detection was studied for detecting small targets in underwater scenes. A multi-scale feature fusion strategy, shortcut feature pyramid network (S-FPN) was proposed to improve the detection accuracy of small targets by introducing various shortcut connections between feature pyramids. Giving an additional detection head to the YOLOv5 model could also enhance the multiple scale detection and improve the detection accuracy of small targets [17]. Attention mechanisms were effective in learning object features and increasing the receptive field could improve the detection accuracy and robustness in recognizing underwater small targets [18]. Furthermore, many works were reported on the modification of feature extraction networks which improved the ability of the feature representation of underwater targets. Residual networks were utilized as a backbone for enhancing the feature extraction efficiency in detecting sea cucumbers [19,20]. Jiang et al. proposed a channel sharpening attention module (CSAM) to further fuse high-level image information [21]. The CSAM was incorporated into the YOLO v3 network and provided the network with the privilege of selecting feature maps. Recently, triplet attention was utilized to modify the YOLO v5 and superior to the state-of-the-art models. Previous works mainly focused on developing or modifying underwater target detection models to achieve better performance in practical applications.

The distortion of underwater images presents different characteristics as natural conditions change, which is a challenge for underwater target detection algorithms to face. Detection of underwater targets from varying underwater scenes is necessary to meet the practical applications of underwater target detection. Image enhancement is a necessary data augmentation method and has been widely used in underwater target detection tasks. A histogram-based image enhancement was reported for the pre-processing of underwater images [22,23]. Huang demonstrated that image enhancement can effectively improve the detection performance of the YOLO v5 in natural scenes [24]. A deep learning-based image restoration was applied to remove haze and light diffusion from the underwater scenes and improved the detection accuracy of sea cucumber [21]. On the other hand, synthetic images are developed as a data augmentation method to enrich the underwater image sets. Huang et al. proposed an improvement of underwater target detection by simulating the images of different marine turbulence environments [25]. Generative adversarial networks (GAN) were utilized to stimulate the image degradation and synthesize images of underwater scenes. The GAN-based approaches could augment the image datasets and improve the detection performance of underwater targets [26].

**Figure 1:**
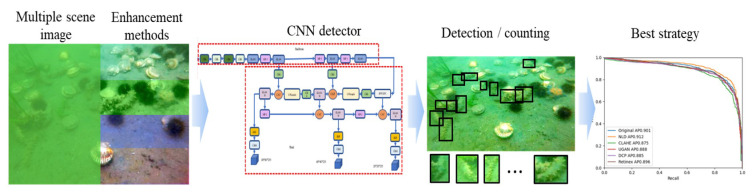
Sea cucumber detection strategy across multiple scenes.

However, marine organism (e.g., sea cucumber) detection across multiple natural scenes has not been widely studied. In addition, the efficiency of image enhancement schemes and the detection accuracy under various distortions were not extensively addressed. Therefore, this work proposes to investigate the sea cucumber detection performance by state-of-the-art object detectors and enhancement methods. The proposed sea cucumber detection strategy is presented in Figure 1. Sea cucumber images from multiple underwater scenes are restored and enhanced by various underwater image enhancement techniques. The enhanced images are trained and tested by the recent object detectors, separately. Finally, the best detection strategy is investigated by analyzing the detection and counting results.

The main contribution of this work is to investigate and analyze the performance of underwater sea cucumber detection in various underwater scenarios using state-of-the-art object detection models and image enhancement.

1. The cutting-edge object detection algorithms, YOLOv7 and YOLO v5, were chosen to implement sea cucumber detection in four different underwater scenes. The experimental results demonstrated that YOLOv7 outperformed YOLO v5, the latest version of faster R-CNN in underwater sea cucumber detection.
2. Frequently applied underwater image enhancement methods, including dehazing models, histogram-based enhancement, physical color model and deep learning-based enhancement, were adopted to test the sea cucumber detection across various underwater scenes. The efficiency of these methods was investigated and the non-local image dehazing (NLD) was the most effective method for all object detection models.
3. Moreover, the real-time ability of YOLOv7 and YOLOv5 was tested. Both of the YOLO models reached the real-time requirements. The proposed work could be a guide for practical underwater surveillance and other related tasks.

The rest of this work is organized as follows: Section 2 describes the details of multiple scene datasets. The proposed sea cucumber detection strategy and the evaluation metrics are presented in Section 3. Experimental setup and results are reported in Section 4. The advantages of the proposed scheme and efficiency of the image enhancement methods are discussed in Section 5. Finally, this work is concluded in Section 6.

## 2. Materials

**Figure 2:**
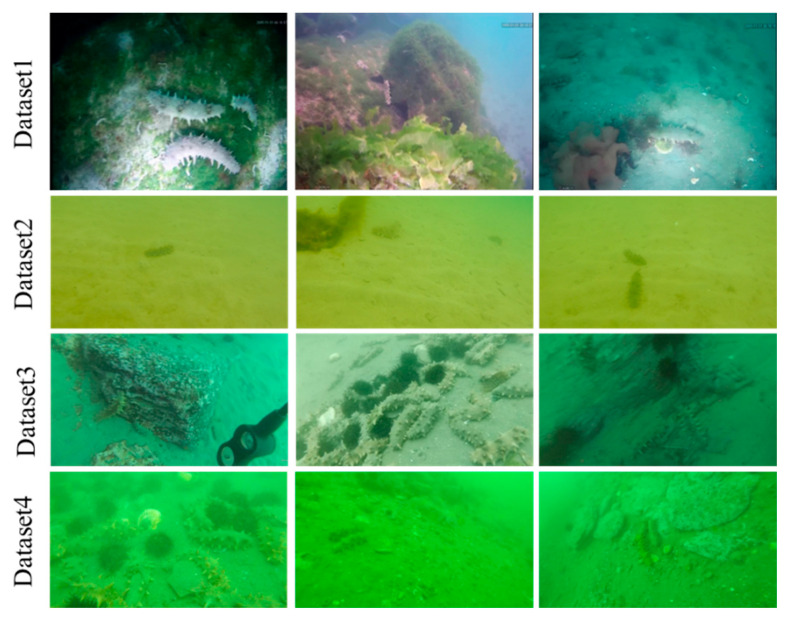
Sea cucumber images in each dataset.

Underwater images from various scenes were collected for developing and verifying the in situ sea cucumber detection scheme. The underwater sea cucumber images were captured by hand-held video cameras (e.g., GoPro) or ROV. Frequently occurring situations in natural conditions are presented in our underwater image dataset, which includes various poses of individual sea cucumbers, varying illuminations, color distortion and image blurring. Images were captured under natural light and artificial lighting. Light conditions present large variation and unequal illumination. Color distortion also occurs under different conditions in our dataset. The image backgrounds contain sea grass, rock and sand. Sea cucumber individuals are shown with a bent body, lying on the ground or climbing rocks. In some places, sea cucumbers aggregate or are occluded by other individuals, grass and rocks. As the underwater images were captured by moving camera, individual sea cucumbers are presented in various scales, and blurred images feature in our datasets. Examples of underwater images in our datasets are presented in Figure 2.

In this work, sea cucumber images are divided into four datasets according to the observation location. Dataset1 contains sea cucumbers on a grass, rock and sand background, and natural illumination and artificial light are both present. Sea cucumbers are scattered in the background, and the individuals are not occluded or attached to each other. Sea cucumber individuals are seldom occluded by grasses. Due to color distortion, images present in blue (natural illumination) and white (artificial light) styles. Dataset2 shows the sandy bottom captured by a diver. Low numbers of sea cucumber individuals are presented in each image, and occlusions are not presented. The image color in dataset2 shows yellow style under natural illumination conditions, and the visibility is sufficiently poor that some individuals are difficult to identify by the naked eye. Sea cucumbers in dataset3 are mainly located on big rocks. Multiple individuals are presented in the images. Some images in dataset3 show aggregated sea cucumbers where individuals are attached to and overlapping each other. In addition, a small proportion of images are blurred due to camera motion. Sea cucumbers are difficult to identify in these images, which are light blue in tone. In dataset4, images are captured among sand and small rocks. Image tone is light green due to color distortion. Blurred images are presented in this dataset.

Detailed information of each dataset is given in Table 1. Sea cucumbers in all datasets were manually labeled by experienced technicians. In this work, 80% of the images were randomly selected as training data, whereas the rest (20%) were used for validation, as in previous studies of deep learning-based computer vision applications [27,28]. Although the image datasets have various image resolutions, all images were normalized to the same according to the requirements for training the detection model. Access to the dataset is given in the Supplementary Materials.

## 3. Methods

### 3.1. Overall Structure

**Figure 3:**
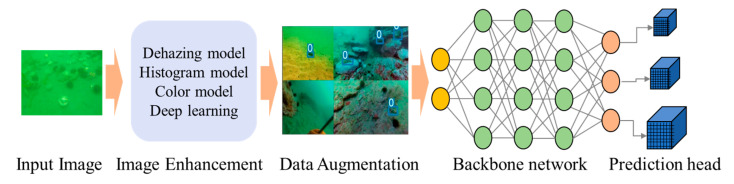
The overall structure of the proposed underwater sea cucumber detection scheme.

The overall structure of in situ sea cucumber detection strategy is illustrated in Figure 3. The proposed in situ detection includes image enhancement, data augmentation, backbone network and prediction head. Five well-known image enhancement methods were utilized to improve the detection accuracy from various underwater scenarios. Data augmentation, such as Mosaic, was adopted to expand the training sets. Image feature of sea cucumbers was extracted by the backbone network. In addition, prediction head identifies the sea cucumber images and estimates their bounding boxes in multiple scales.

### 3.2. Object Detection by YOLOv7

The YOLO series of object detection model is a classical one-stage prediction framework which processes object detection and classification in a single neural network [29]. Object detection can be conducted in milliseconds by YOLO. The YOLO framework is composed of input module, backbone, neck and detection heads [30]. The input image is preprocessed in the input module and the backbone is the convolutional neural network that extracts and aggregates image features with different image levels. The neck deals with multiple scale processing and delivers various scales of features into the detection head for detection of various sized objects. Finally, the detection head generates bounding boxes for each object and predicts their classes.

**Figure 4:**
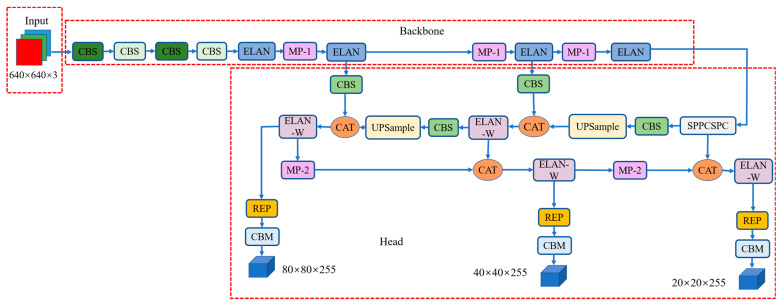
The network architecture of YOLOv7 (“CAT” indicates concatenation operation).

The network structure of YOLOv7 is illustrated in Figure 4. YOLOv7 consists of input, backbone, and prediction head [31]. In YOLOv7, the neck part is merged into the prediction head to deal with multiple scale object detection.

In the input layer of YOLOv7, mosaic data augmentation is adopted to enrich the image features of targets. Mosaic augmentation combines four images by randomly scaling, cropping, and re-arranging them. The labelled targets are much increased. Mosaic augmentation has been demonstrated to be effective in detecting small targets, such as in underwater scenes. YOLOv7 adopts image resolutions of 608 × 608 pixels and 1280 × 1280 pixels as the input of the network. Due to the different aspect ratios of input images, the sizes of black edges at both ends are different after scaling and filling.

**Figure 5:**
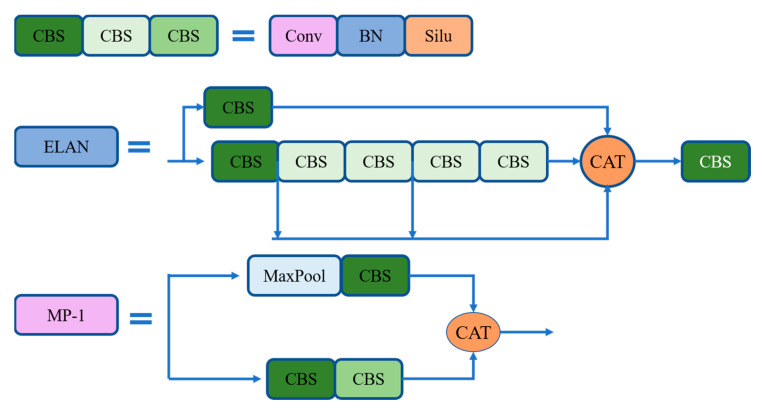
The CBS structure, ELAN structure, and MP-1 structure of backbone.

Backbone of YOLOv7 is the combination of CBS module, ELAN module and MP-1 module. The structure of CBS, ELAN, and MP-1 of backbone are shown in Figure 5. The CBS module consists of convolution, batch normalization and SILU activation functions. The ELAN module is the concatenation of multiple CBS modules. This module enables deeper networks to learn and converge by controlling the shortest and longest gradient paths. The MP-1 structure is the concatenation of two CBS branches. The upper branch consists of MaxPool and CBS, and the lower branch contains two CBS modules.

**Figure 6:**
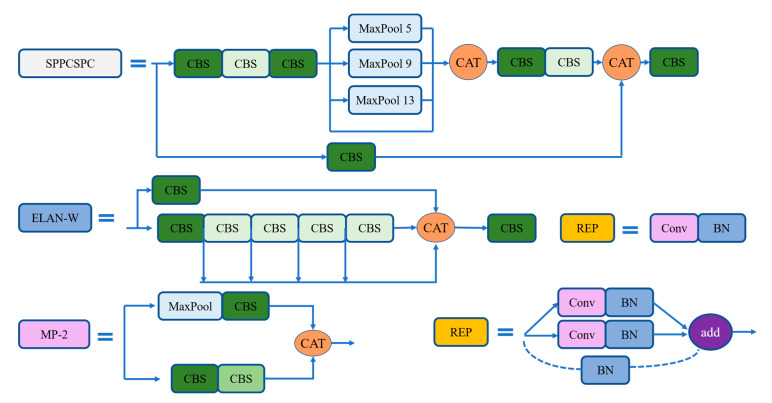
The SPPCSPC structure, ELAN-W structure, REP structure and MP-2 structure of head.

The head layer of YOLOv7 consists of SPPCSPC structure, ELAN-W structure, MP-2 structure and RepVGG block. The SPPCSPC structure, ELAN-W structure, REP structure and MP-2 structure of the head are shown in Figure 6. The structure of PepVGG block is different during training and deployment. During training, it is composed of a 3 × 3 convolution and a 1 × 1 convolution branch. If the channels, length and width of the input and output are the same, another BN branch will be added, and the three branches will be added as the output. During the deployment, to facilitate the deployment, the parameters of the branch will be re-parameterized to the main branch, and the convolution output of the main branch of 3 × 3 will be taken. The head layer finally outputs three different sizes of unprocessed predictions through the three RepVGG and Conv layers.

### 3.3. Image Enhancement Methods

In general, image enhancement is utilized to emphasize the global or local features of an image, such as improving the color representation, brightness and contrast of an object. Image enhancement is widely applied to improve the clarity of images, emphasizing certain features of interest, enlarging the differences between objects and backgrounds and suppressing uninteresting features. Therefore, image enhancement has been commonly used in underwater image processing and target detection. In this work, five well-known image enhancement methods are chosen to evaluate the efficiency of image enhancement in underwater sea cucumber detection. These image enhancements are contrast limited adaptive histogram equalization (CLAHE), dark-channel prior (DCP), non-local image dehazing (NLD), Retinex, and underwater generative adversarial network (UGAN), which covers histogram-based method, dehazing method, physical color model, and deep learning-based method.

Contrast limited adaptive histogram equalization is a variant of adaptive histogram equalization (AHE). CLAHE can reduce the noise problem of AHE by limiting contrast enhancement [32]. It calculates multiple histograms and each of them corresponds to a different part of the image. The brightness of the image is redistributed according to these histograms. CLAHE limits the amplification by clipping the histogram at a user-defined value called clip limit. The clipping level determines how much noise in the histogram should be smoothed and hence how much the contrast should be enhanced. Thus, CLAHE is suitable for enhancing the local image contrast and emphasizing edge features in each part of the image.

Dark-channel prior is a statistical rule for haze-free images. He et al. found that there are always pixels with at least one intensity value that is close to zero within an image patch [33]. In the process of dark channel extraction, the image is decomposed in RGB space, and the minimum value operation is taken in the local block to obtain the minimum component in the three channels (R, G, B). A Marcel Van Herk’s algorithm is used to implement the local region minimum filtering on the minimum component value, i.e., the gray level corrosion operation. The effectiveness of DCP in dehazing is proved by its applications in solving haze removal issues.

Non-local image dehazing assumes that colors of a haze-free image are well approximated by a few hundred distinct colors that form tight clusters in RGB space and pixels in a cluster are often non-local [34]. The term haze-line is proposed to estimate the transmission factors. In this method, clustering is used to group the pixels so that each cluster becomes a haze-line. Then, the maximum radius of each cluster is calculated and used to estimate the transmission. A final regulation step is performed to enforce the smoothness of the transmission map. The NLD could improve the visibility and enhance the detailed image features. 

Retinex is a composite of retina and cortex and is referred to as the retinal cortex theory. The basic idea of Retinex theory is that the illumination intensity determines all pixels in the original image, and the inherent property of the original image is determined by the reflection coefficient of the object itself. That is, the reflection image and the illumination image are assumed to be the original image. Therefore, Retinex is to remove the influence of illumination and retain the inherent property of the object [35].

Recently, the generative adversarial network (GAN) presented outstanding performance in image synthesis and style transferring. The underwater GAN (UGAN) uses an adversarial approach towards generating realistic underwater images. UGAN structures the problem of estimating the real appearance of underwater imagery as a paired image-to-image translation problem [26]. In the training process, UGAN learns the restoration model from the image pairs taken in two independent domains (e.g., underwater and ground).

**Figure 7:**
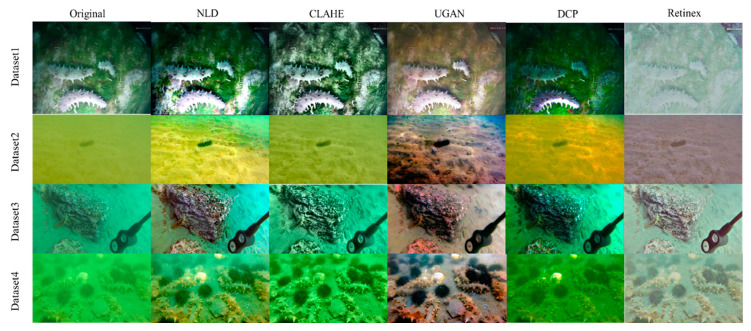
Examples of underwater image datasets and image enhancement.

Examples of enhanced images of our datasets are illustrated in Figure 7. The selected image enhancement methods present various characters of images in different scenes. The enhanced image sets are used to train the object detection models and to evaluate the efficiency of each enhancement method for detecting sea cucumbers.

### 3.4. Evaluation Metric

Common metrics of object detection are adopted to evaluate the performance of sea cucumber detection. The individual detection results are evaluated and compared by the precision–recall analysis and average precision (AP) [36,37]. The precision–recall analysis is conducted by calculating true positive (TP), false positive (FP) and false negative (FN). True positives are the detected results which match the ground truth. False positives are the results reported by the detection algorithm but are actually incorrect. In other words, TP contains sea cucumber individuals, whereas FP has no sea cucumbers. Usually, background objects are confused with the detection targets due to appearance similarity or inaccurate detectors. In object detection, the target objects which cannot be identified by the detection algorithm are counted as false negatives. The precision measures the proportion of correct results from the total detection results. High precision indicates the detection results containing a high percentage of reliable results and a low percentage of false alarms. Precision is calculated from TP and FP (Equation (1)). Recall represents the detection accuracy of sea cucumbers and refers to the percentage of correctly detected individuals from the total number of sea cucumbers (Equation (2)). To evaluate the overall performance of object detection, F measure is calculated by considering both precision and recall (Equation (3)). A high F measure score indicates that the detection results are accurate and reliable.

(1) $$\mathrm{Precision}=\frac{\mathrm{True}\;\mathrm{Positive}}{\mathrm{True}\;\mathrm{Positive}+\mathrm{False}\;\mathrm{Positive}}$$

(2) $$\mathrm{Recall}=\frac{\mathrm{True}\;\mathrm{Positive}}{\mathrm{True}\;\mathrm{Positive}+\mathrm{False}\;\mathrm{Negative}}$$

(3) $$F=\frac{2\cdot \mathrm{Precision}\cdot \mathrm{Recall}}{\mathrm{Precision}+\mathrm{Recall}}$$

Average precision (AP) is a widely applied metric for evaluating object recognition/detection [37]. Average precision calculates the shape of the precision/recall curve and replaces the area-under-curve (AUC) of ROC curve to improve the sensitivity of the metric. Average precision is defined as the mean precision at 11 equally divided levels in recall [0, 0.1, …, 1]. The calculation of AP is given in Equation (4):

(4) $$\mathrm{AP}=\frac{1}{11}\sum_{r\in \left\{0,0.1,\ldots ,1\right\}}P_{interp}(r)$$

where *P_interp_* represents the interpolated precision at a certain recall level *r*. Another term for evaluating detection accuracy is IOU. This measures the area of overlap *a_o_* between the detected bounding box *B_p_* and ground truth bounding box *B_gt_*. Intersection over union evaluates the accuracy of predicted bounding box:

(5) $$a_{o}=\frac{area\left(B_{p}\cap B_{gt}\right)}{area\left(B_{p}\cup B_{gt}\right)}$$

where *B_p_* ∩ *B_gt_* is the intersection of the two bounding boxes and *B_p_* ∪ *B_gt_* is the union of them. Usually, a threshold of 50% of IOU is required to examine the detection results.

## 4. Experiments and Results

### 4.1. Experiment Conditions

Sea cucumber detection models were trained on original images and enhanced images separately. The same model structure and training parameters were adopted in our experiments. Image size was normalized to 608 × 608 pixels and 640 × 640 pixels for all tests by YOLOv7 and YOLO5, respectively. The initial learning rate was set to 0.01 and batch size was set to 4 by considering the memory limitation of the graphic card. All models were trained for 100 epochs until the training loss presenting convergence. These parameters were determined according to previous studies [38] and our preliminary tests for both YOLOv7 and YOLOv5. In addition, the latest version, Faster R-CNN, provided by PyTorch, was employed to conduct the comparison tests. All experiments were carried out on a NVidia Titan V Graphics Processing Unit (GPU) with 12G Video Random Access Memory (VRAM). The software environments were configured by Ubuntu 16.04 and PyTorch 1.7.

### 4.2. Model Training

**Figure 8:**
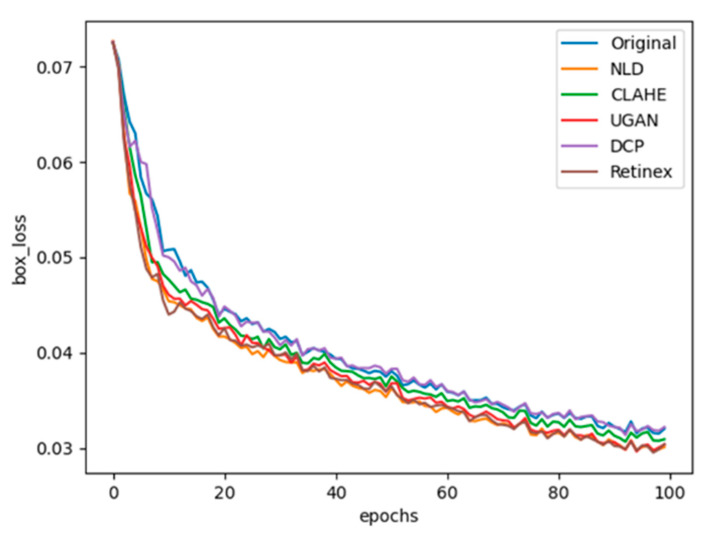
Train losses of image enhancement methods in YOLOv7.

In the training process, all images from the training datasets were adopted to train the sea cucumber detection model. Training losses were recorded at each epoch. According to the preliminary tests the maximal epoch was set to 100. The training loss curves of YOLOv7 are presented in Figure 8 and the losses for each method are distinguished by colors. The training loss of YOLOv7 decreased faster with image enhancements. Especially, NLD, Retinex and UGAN could significantly decrease the loss values and enhance the learning efficiency of the YOLOv7 model.

### 4.3. Experimental Results

Experimental results of sea cucumber detection using YOLOv7 and enhanced images are presented in Table 2. Average precision at IOU ≥ 0.5 (AP_50_) is adopted to evaluate the detection performance of the proposed methods for each test dataset in this work (Hereafter, AP denotes AP_50_ for short). The effectiveness of each image enhancement method was evaluated by combining with YOLOv7. The accuracies of sea cucumber detection by each combination were tested on the four scenes in our datasets, separately. Accordingly, the averaged accuracy from all the testing sets was calculated to demonstrate the performance of each detection scheme across different scenes. In Table 2, the best results in each column are marked in bold and the second-best results are given in bold italics. On average, the best AP across all datasets reached 0.94, which was achieved by YOLOv7 with NLD enhancement and the original YOLOv7 was the second best (0.929) from all datasets. The AP was improved by 1.1% by NLD enhancement compared with YOLOv7 without enhancement (0.929). The NLD enhancement had the best APs in datasets 1, 3 and 4. Especially, the AP of NLD reached 0.948 in dataset 1 and was 4.0% higher than the YOLOv7. Moreover, CLAHE and Retinex were also outperformed the YOLOv7 in dataset1. 

To evaluate the effectiveness of image enhancement and performance comparison, YOLOv5 was further employed for conducting the tests with image enhancements in multiple scenes. The experimental results by YOLOv5 and image enhancement are presented in Table 3. From the average values of AP of the four datasets, the highest AP is 0.928, which was the result of YOLOv5 with NLD image enhancement. The detection result of the original image has the second highest AP of 0.912. The results showed that the NLD enhancement improved the AP of YOLOv5 by 1.6%. The NLD image enhancement achieved the best performance in datasets 1, 3 and 4, which is consistent with the effect of NLD image enhancement in YOLOv7. Although detection with NLD was not the best result in dataset2 it still improved the AP by 1.9% compared with the original YOLOv5. Therefore, YOLOv5 with NLD obtained the best AP across all scenes. These results indicate that NLD image enhancement can improve the detection accuracy in multiple scenes. The best detection in scene two is with CLAHE and DCP image enhancement. The results showed that CLAHE and DCP were effective and stabile in simple scenes.

Furthermore, the overall detection performance was evaluated on YOLOv7, YOLO v5 and faster R-CNN (Table 4). It is notable that detection accuracy was significantly improved by YOLOv7 and YOLO v5 compared with the faster R-CNN model. The detection accuracy by YOLOv7 with NLD was 9.3% higher than the faster R-CNN without enhancement. Faster R-CNN is widely adopted as baseline model to implement and evaluate the underwater object detection. The proposed work showed better improvement on sea cucumber detection compared with recent works [22,39,40]. The experimental results indicate YOLOv7 and NLD are effective and promising in real applications, such as automatic sea cucumber monitoring. 

Precision–recall analysis was further conducted to investigate the performance of each method. The precision, recall and F score of YOLOv7 combined with image enhancements are shown in Table 5. Overall, YOLOv7 with NLD achieved the highest F score of 0.902 from all datasets and was 2.3% higher than YOLOv7 without enhancement (0.879). The second best is the CLAHE enhanced result of 0.887. The F scores of Retinex and DCP were followed and were slightly higher than the YOLOv7. The UGAN showed a similar level with YOLOv7 and could not improve the F score for multiple scene detections. From the four scenes, the CLAHE and DCP presented best recall of 0.881; however, their precision values were much decreased. The best precision was obtained by NLD enhancement (0.928) which was improved by 1.4% comparing with YOLOv7 without enhancement.

Moreover, precision and recall analysis of YOLOv5 are presented in Table 6. The highest F score for all datasets was 0.896 which was the result of NLD enhancement. It was 1.14% increased from the YOLOv5 without enhancement. However, the other image enhancements showed lower F scores compared to the detection by only YOLOv5. That means CLAHE, UGAN, DCP and Retinex were less effective for YOLOv5 in detecting sea cucumber across multiple underwater scenes. These methods could be effective in specific scenes. For example, Retinex obtained the best F score of 0.823 in dataset3 and DCP presented an F score of 1.0 in dataset2, which were much improved from the original YOLOv5. Moreover, the precision and recall values produced by NLD enhancement were 0.925 and 0.871, respectively. The NLD showed the best recall and the second-best precision values. The DCP presented the highest precision of 0.939 for all datasets and was 1.4 higher than the NLD enhancement.

**Figure 9:**
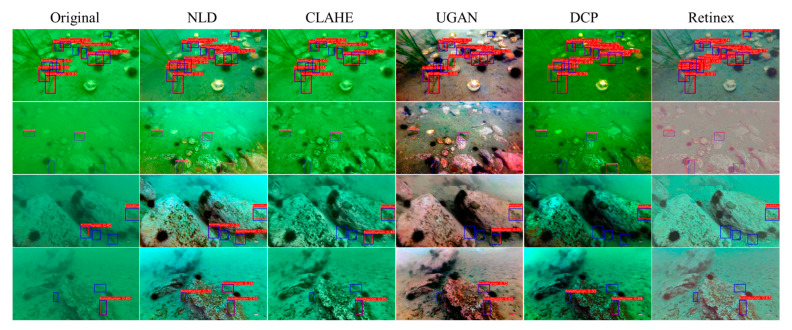
Examples of detection results by YOLOv7 with image enhancements.

A qualitative evaluation of sea cucumber detection is presented in Figure 9. Examples of detection results by YOLOv7 explain the effectiveness of each image enhancement method. In Figure 9, blue boxes indicate ground truth and the detection results are marked by red boxes. False positives seldomly occurred in the results. The detection results by NLD enhancements contained the most true positives since the enhanced images presented better contrast. Although the images enhanced by UGAN showed the best color restoration for all scenes, the detection accuracy was not as high as we expected because the sea cucumber images are dark and the detailed features (e.g., texture of sea cucumber body) were depressed after enhancement. The other enhancement methods also could not improve the detection rates for all cases.

In addition to detection accuracy, time consumption of YOLOv7 and YOLOv5 for prediction on the original image dataset and enhanced datasets are analyzed in Table 6. The average time for the prediction of a single image of all the datasets was calculated for each detection model combined with image enhancement. The average prediction time was 4.3 ms and 6.2 ms for YOLOv7 and YOLOv5, respectively. On average, YOLOv7 was approximately 30% faster than YOLOv5. The prediction time for each image enhancement was almost the same since the tests were run on the enhanced image datasets. The time consumption of image enhancement was not included in our tests. Table 7 shows that the enhanced image hardly affects the prediction time, which is equal to or slightly less than the prediction time required for the original images.

## 5. Discussion

Detecting sea cucumbers in natural conditions is a challenging task, especially in complex scenes with low-quality images. The outstanding ability of feature extraction by deep convolutional neural network and effectiveness of image enhancement were verified in this work. The latest version of YOLOv7 was effective in detecting sea cucumbers in various conditions. In the experiments, the AP of YOLOv7 was 1.7% higher than the AP of YOLOv5, which proved the advancement of YOLOv7. The NLD could effectively increase the detection accuracy of sea cucumber across multiple scenes for both YOLOv7 and YOLOv5. The APs of YOLOv5 on each dataset were improved by applying NLD enhancement (Table 3). For YOLOv7, the APs of dataset1, 3 and 4 were successfully increased by applying NLD enhancement. The improvement of detection performance by NLD is attributed to the enhancement of detailed or local image features and image contrast. Estimating and removing haze lines was suitable for the application of underwater target detection. On the contrary, CLAHE, DCP, UGAN and Retinex could not contribute improvements for sea cucumber detection in all datasets. These image enhancement methods could only improve the detection performance in a certain scene. For example, the APs of YOLOv5 enhanced by CLAHE and DCP reached the best AP of 0.995 in dataset2. In addition, CLAHE and Retinex could significantly improve the AP of YOLOv7 in dataset1. Although the images reconstructed by UGAN present the best color restoration to the human vision system (Figure 5) it could not help to improve the detection accuracy in the tests. The results indicate that color restoration is less effective to enhance the detection ability of YOLOv7 and YOLOv5 since a series of data augmentation methods (e.g., mosaic) is utilized to enrich the feature representation in YOLOv7 and YOLOv5 and the detection models focus on learning morphological and texture features of the targets.

One of the challenging issues in sea cucumber detection is the clarity of images. The images in dataset1 and dataset2 presented distinct appearance features, and sea cucumber individuals showed regular shape and less occlusion. These images were clear, with high resolutions. Although color distortion occurred in dataset1 and dataset2, the body feature of the sea cucumber was distinct from the background. The deep convolutional network accurately learned the shape of the sea cucumber. Therefore, high values of AP in dataset1 and dataset2 infer those images presenting typical sea cucumber characteristics (e.g., the “prickle” on the sea cucumber’s body) could be accurately recognized even under slight color distortion, whereas the image enhancement could improve the detection accuracy from low-quality images. Small-sized objects could be accurately detected by enhancing the image quality. These small sea cucumbers were usually difficult to find in the original image due to low visibility. After dehazing processing, the image feature and contrast of these small objects could be emphasized. Consequently, they could be accurately identified from the background. On the contrary, dataset3 and dataset4 presented rather difficult situations. Image blurring was severe and visibility was low. Many of the individuals were also difficult to identify for human experts. Therefore, the AP of YOLOv5 in dataset3 and dataset4 were much lower than dataset1 and dataset2. It is notable that the detection accuracies of dataset3 and dataset4 were significantly improved by YOLOv7, especially as the AP was improved by 4.2% by combing YOLOv7 and NLD enhancement in dataset4 (Table 2 and Table 3).

In addition to the poor-visibility issue in underwater images, image blurring is another main factor decreasing detection accuracy in underwater images. The blurred image is caused by the fast motion of the cameras. In this research, images in dataset3 and dataset4 were the frames from underwater videos captured by a ROV. The high-speed movement or rotation of ROV could lead to image blurring, and the undercurrent in the water could make ROV shaky or spinning, and consequently blurred images were recorded in this situation. In fact, the blurred sea cucumber images did not show much “sea cucumber features”. On the other hand, sea grass and rocks presented similar appearance in the low-quality underwater images. Most false alarms incorrectly identified the brown-colored sea grasses and small stones as sea cucumbers. When the visibility was low, the detection model also located the texture on a rock surface as sea cucumber due to texture similarity. To improve the stability of sea cucumber detection, recognition using image sequence could be considered to reduce the effects of blurred or less visible images. 

## 6. Conclusions

An underwater sea cucumber detection scheme was implemented based on the state-of-the-art object detection frameworks and image enhancements. The detection performance of YOLOv7, YOLOv5 and their combinations with five image enhancement methods were investigated. Experimental results showed that YOLOv7 enhanced by non-local image hazing achieved the best AP of 0.94 which was superior to YOLOv7 and YOLOv5. YOLOv7 outperformed YOLOv5 in AP and precise-recall analysis that proved YOLOv7 is accurate and reliable for object detection (e.g., sea cucumber) in various natural conditions. Moreover, the non-local image hazing was the most effective image enhancement method for improving performance of sea cucumber detection in multiple underwater scenes. The results demonstrated that non-local image hazing could improve the detection accuracy for YOLOv7, YOLOv5 and faster R-CNN. Furthermore, the inference times of YOLOv7 and YOLOv5 were examined and real-time performance was reached.

The investigated results in this work could be a guidance for underwater target detection and image process task. The proposed scheme could be a solution for practical underwater survey for aquaculture products. For future research, the proposed method should be applied to practical tasks on underwater surveillance or integrated to underwater mobile vehicles.

## Figures and Tables

**Table 1:** Details of image datasets in this study.

	No. of Images	No. of Individuals	Image Resolution
Dataset1	123	412	1024 × 576
Dataset2	76	133	1920 × 1080
Dataset3	1095	2678	720 × 405
Dataset4	392	1078	720 × 405
Total	1686	4301	

**Table 2:** Average precision (AP_50_) of sea cucumber detection at multiple scenes by YOLOv7 (Bold font indicates the best result and italic indicates the second-best result).

Enhancement Methods	Dataset1	Dataset2	Dataset3	Dataset4	Average
Original	0.908	**0.994**	***0.878***	***0.936***	0.929
NLD	**0.948**	0.985	**0.884**	**0.944**	**0.940**
CLAHE	***0.939***	**0.994**	0.843	0.908	0.921
UGAN	0.901	0.991	0.869	0.911	0.918
DCP	0.907	0.992	0.861	0.913	0.918
Retinex	0.935	0.989	0.868	0.930	***0.930***

**Table 3:** Average precision (AP_50_) of sea cucumber detection at multiple scenes by YOLOv5 (Bold font indicates the best result and italic indicates the second-best result).

Enhancement Methods	Dataset1	Dataset2	Dataset3	Dataset4	Average
Original	***0.930***	0.967	0.851	***0.902***	***0.912***
NLD	**0.953**	0.989	**0.863**	**0.908**	**0.928**
CLAHE	0.922	**0.995**	0.805	0.889	0.903
UGAN	0.824	***0.990***	0.827	0.883	0.881
DCP	0.870	**0.995**	0.852	***0.902***	0.905
Retinex	0.833	0.984	***0.854***	0.894	0.891

**Table 4:** Performance evaluation (AP_50_) of YOLOv7, YOLO v5 and faster R-CNN.

	Original	NLD	CLAHE	UGAN	DCP	Retinex
Faster R-CNN	0.847	0.854	0.814	0.828	0.835	0.838
YOLO v5	0.912	0.928	0.903	0.881	0.905	0.891
YOLOv7	0.929	0.940	0.921	0.918	0.918	0.930

**Table 5:** Precision–recall analysis of YOLOv7 with image enhancement (Bold font indicates the best result and italic indicates the second-best result).

	Dataset1			Dataset2			Dataset3			Dataset4			Average		
	P	R	F	P	R	F	P	R	F	P	R	F	P	R	F
Original	0.947	0.766	0.847	***0.958***	**1.000**	**0.979**	0.824	0.803	0.813	***0.927***	0.832	***0.877***	***0.914***	0.850	0.879
NLD	**0.976**	0.872	***0.921***	0.920	**1.000**	0.958	**0.873**	0.781	**0.824**	**0.944**	**0.864**	**0.902**	**0.928**	0.879	**0.902**
CLAHE	0.935	**0.915**	**0.925**	***0.958***	**1.000**	**0.979**	0.806	0.788	0.797	0.871	0.822	0.846	0.893	**0.881**	***0.887***
UGAN	0.840	0.893	0.866	**1.000**	0.956	0.978	***0.835***	0.795	***0.815***	0.855	0.855	0.855	0.883	0.875	0.878
DCP	0.857	***0.894***	0.875	***0.958***	**1.000**	**0.979**	0.818	***0.812***	***0.815***	0.916	0.818	0.864	0.887	**0.881**	0.883
Retinex	***0.951***	0.829	0.886	0.919	**1.000**	0.958	0.805	**0.821**	0.813	0.893	***0.859***	0.876	0.892	0.877	0.883

**Table 6:** Precision–recall analysis of YOLOv5 with image enhancement (Bold font indicates the best result and italic indicates the second-best result).

	Dataset1			Dataset2			Dataset3			Dataset4			Average		
	P	R	F	P	R	F	P	R	F	P	R	F	P	R	F
Original	0.907	0.830	*** 0.867 ***	** 1.000 **	0.956	*** 0.978 ***	0.819	** 0.804 **	*** 0.811 ***	0.896	** 0.846 **	** 0.870 **	0.906	*** 0.859 ***	*** 0.882 ***
NLD	0.936	** 0.936 **	** 0.936 **	** 1.000 **	0.957	*** 0.978 ***	0.826	0.795	0.810	** 0.939 **	0.794	*** 0.860 ***	*** 0.925 ***	** 0.871 **	** 0.896 **
CLAHE	0.840	*** 0.894 ***	0.866	** 1.000 **	0.957	*** 0.978 ***	0.830	0.741	0.783	*** 0.934 ***	0.724	0.816	0.901	0.829	0.861
UGAN	** 0.971 **	0.723	0.829	0.920	** 1.000 **	0.958	** 0.892 **	0.703	0.786	0.850	*** 0.822 ***	0.836	0.908	0.812	0.852
DCP	** 0.971 **	0.723	0.829	** 1.000 **	** 1.000 **	** 1.000 **	0.849	0.766	0.805	*** 0.934 ***	0.790	0.856	** 0.939 **	0.820	0.873
Retinex	0.919	0.723	0.809	0.999	0.913	0.954	*** 0.850 ***	*** 0.797 ***	** 0.823 **	0.883	0.813	0.847	0.913	0.812	0.858

**Table 7:** Prediction time of YOLOv7 and YOLOv5 (unit: ms).

	Original	NLD	CLAHE	UGAN	DCP	Retinex	Average
YOLOv5	6.3	6.1	6.3	6.3	6.2	6	6.2
YOLOv7	4.5	4.5	4.3	4.2	4.2	4.1	4.3

## Data Availability

Data will be made available on request.

## Supplementary Materials

Image datasets used in this work can be found online at: https://github.com/chunleixia/seacucumberdata (accessed on 20 December 2022).
